# 

**Published:** 1912-01

**Authors:** 


### CONTENTS.

ORIGINAL COMMUNICATIONS:—

Etiology, Pathology and Treatment of Pellagra. By Geo. C. Mizell, D. D. ...... 523

Neurologic Diagnosis—Some First Principles. By Hansell Crenshaw, M. D., Atlanta, Ga. .......... 540

The Technique of Prostatectomy. By Edgar G. Ballenger, M. D., and Omar F. Elder, M. D., Atlanta, Ga. .......... 543

EDITORIALS .......... 551

NEWS AND NOTES .......... 555

BOOK REVIEWS .......... 573

